# Biology, Pathogenicity, and Genetic Diversity of the Rice Pathogen *Ustilaginoidea virens* in Heilongjiang Province, China

**DOI:** 10.3390/biology14010046

**Published:** 2025-01-09

**Authors:** Peng Guo, Xiaofeng Xu, Yue Ma, Niaz Nihal, Mingxiu Yang, Zhe Ni, Younis Haseeb, Lei Hou, Anqi Lv, Junhua Zhang

**Affiliations:** College of Plant Protection, Northeast Agricultural University, Harbin 150030, China; a395190874@163.com (P.G.); oxuxiaofeng@163.com (X.X.); 18846835264@163.com (Y.M.); nihalniaz@hotmail.com (N.N.); 2002ymx@163.com (M.Y.); nizhe_888@163.com (Z.N.); haseebyounis99@gmail.com (Y.H.); leileilulala@163.com (L.H.); l928446289@163.com (A.L.)

**Keywords:** biological characteristics, genetic diversity, Rep-PCR, rice false smut, screening of tolerance materials, *Ustilaginoidea virens*

## Abstract

Rice false smut (RFS) is pervasive and has emerged as the primary disease affecting rice productivity. Due to the lack of effective chemical control, disease-resistant varieties are the primary method of managing the disease. This study aimed to investigate the influence of biological characteristics such as hyphal growth rate, spore production and germination ability on the pathogenicity of *Ustilaginoidea virens*. The genetic diversity of 86 *U. virens* isolates was analyzed, and the tolerant varieties were identified using Rep-PCR molecular markers. The results indicated that among the *U. virens* isolates analyzed, about 31, 27 and 28 strains exhibited fast, medium and slow mycelium growth rates, respectively. Among the *U. virens* isolates analyzed, about 33, 27 and 26 strains exhibited strong, medium and weak sporulation abilities, respectively. In addition, among the *U. virens* isolates analyzed, about 29, 30 and 27 strains exhibited strong, medium and weak spore germination, respectively. No significant correlation was noted between mycelium growth rate and pathogenicity, but sporulation ability, spore germination ability and liquid culture were positively correlated (*p* < 0.05). Moreover, the genetic structure of *U. virens* was diverse and complex. There was a significant correlation between the genetic differentiation and pathogenicity of *U. virens* in Heilongjiang Province, China, with a maximum coefficient of 0.1786 and a minimum coefficient of 0.0129 between Harbin and Suihua, but no significant correlation was found with gene groups or geographical regions. The study found that genes and geographical population variation were primarily within the population, with 95.63% and 4.37% variation (*p* > 0.05), respectively, while pathogenicity population variation was mainly within the population, with 86.08% and 13.92% variation (*p* > 0.05), respectively. Pathogenicity had a significant effect on the differentiation of *U. virens*. Most rice varieties were susceptible, with 3 resistant, 8 moderately resistant, 9 moderately susceptible, 8 susceptible and 12 highly susceptible to RFS disease. Collectively, these results indicate that the tolerance of rice against *U. virens* is poor, which merits further investigation.

## Simple Summary

Rice false smut (RFS) is a major threat to rice productivity, and disease-resistant varieties are crucial as chemical control is ineffective. This study explored how the biological features of *Ustilaginoidea virens* affect its pathogenicity. The genetic diversity of 86 isolates was analyzed, and tolerant rice varieties were identified. Results showed different growth rates, sporulation and spore germination levels among isolates. For sporulation and spore germination, there was no correlation between the mycelium growth rate and pathogenicity. The genetic structure of *U. virens* is complex. Pathogenicity impacts its differentiation. Most rice varieties are susceptible. Overall, rice’s tolerance to *U. virens* is poor and needs more study.

## 1. Introduction

Rice (*Oryza sativa* L.) is the most significant agricultural crop in the developing world. Rice security is not merely an economic concern but also a key factor in determining social and political stability [1,2]. Thus, rice research must be focused on developing ways to mitigate insect and disease losses. Several minor rice diseases have assumed major disease status during the last few decades, mainly due to adoption of new cultivation practices and introduction of new hybrids and varieties [3]. Rice false smut (RFS) is a disease caused by *U. virens*, a species within the Ascomycota phylum and Ustilaginaceae family. It leads to a severe grain disease in rice. It has emerged as one of the most significant threats to productivity and grain quality in rice-growing countries globally [2].

RFS has historically been regarded as a minor rice disease with sporadic occurrences in several rice-growing regions like China, India and the USA [4,5]. Since the 1970s, due to the massive planting of high-yield rice cultivars and hybrids, incorrect use of synthetic fertilizer and global warming, the prevalence of RFS has grown year after year in China and has become a major rice disease from a minor disease [2,6]. The causative agent of RFS is an ascomycete fungal pathogen, *U. virens* (Cooke) Takah (teleomorph: Vil-losiclava virens) [7]. In China, RFS has an average annual incidence area of 3.06 million ha, resulting in a yield loss of 158.6 million kg year^−1^. From 2008 to 2016, the annual average area of prevention and control for this disease was 6.92 million hectares [8]. In particular, 19–40% of the rice-growing region in the middle and lower portions of the Yangtze River is severely affected by RFS disease [8]. It has been reported that in India, false smut-infected tillers varied from 5% to 85%, resulting in a yield loss of 0.2–49% depending on rice variety and disease severity [9,10]. There have also been reports of RFS outbreaks in the Middle East and North America [11,12].

*Ustilaginoidea virens* infects rice flowers [13] and colonizes the interior organs with mycelia, transforming them into false smut balls coated in powdered chlamydospores [14]. False smut balls range from yellowish orange to greenish black [2] and frequently produce sclerotia when confronted with considerable temperature variations between day and night [15]. RFS not only prevents rice grain filling but also increases the sterility of spikelets near the balls, resulting in a significant loss in grain yield [16]. RFS disease outbreaks are usually associated with rainfall during the rice booting and heading phases, with epidemics varying greatly by variety, field and season. In addition to the apparent yield loss [17], it can create considerable quantities of toxins, such as ustiloxin, ustilaginoidin and sorbicillinoids, which are hazardous to both people and animals [18]. They may disrupt a variety of physiological processes, including hormone regulation, nutrient intake and photosynthesis, resulting in reduced plant growth and grain yield. It may inhibit plants from getting the necessary nutrients from the soil [5,10]. The prevalence of RFS disease incidence is significantly increasing, affecting rice yield and quality in Heilongjiang province [4]. Thus, an understanding of the *U. virens* disease cycle and infection process, as well as nutrition acquisition and disease prevention and control strategies, is of paramount importance.

Understanding the morphological characterization of the pathogens will provide a theoretical basis to build optimal disease control strategies [8]. The *U. virens* from different geographical regions and in different rice cultivars show diverse characteristics. When cultured in media, they produce various colony and chlamydospore features. For most strains of *U. virens*, the colony color changes according to the maturity time. Initially, it is white. Then, it turns yellow and finally becomes green [19]. The rate of mycelial growth is variable, as the growth patterns range from very slow to slow, moderate and fast [5].

Reducing the severity of the disease depends on an integrated approach to disease management. The first steps in preventing and managing the disease include selecting seeds, taking into account suitable cultural practices and selecting an effective control agent [2,4]. Breeding and using resistant cultivars are the most efficient and inexpensive strategies to manage RFS disease and assure excellent yields of rice [20]. Moreover, the most cost-effective and efficient way to manage RFS is to choose resistant varieties [7].

The widespread cultivation of a single rice variety encouraged mutations in rice fungus and reduced the crop’s resistance, resulting in a yearly increase in the prevalence of RFS disease. Under this agricultural production model, pathogens evolve towards stronger pathogenicity and faster transmission speeds. Changes in the population structure of pathogens affect the occurrence, prevalence and control of diseases. The genetic structure analysis of *U. virens* can explore issues such as the transmission routes, centers of origin, genetic diversity and genetic migration of *U. virens* in different years and regions [21]. This is significant for cultivating rice varieties resistant to rice false smut and controlling the disease. How to effectively prevent and control RFS disease is an important concern that must be resolved. In this study, the impacts of biological features of 86 strains of *U. virens* isolated and purified in Heilongjiang province were examined, and the correlation between mycelium growth rate, sporulation ability, spore germination, liquid culture color and pathogenicity was evaluated. Rep-PCR molecular marker technology was utilized to study the genetic diversity of *U. virens*, identify the genetic differences and similarities and screen for tolerant varieties, providing a theoretical basis for the prevention and control of RFS, rational planting of rice varieties and breeding of resistant varieties. So far, there have been few studies on *U. virens* causing RFS, and the relationship between biological characteristics and pathogenicity, genetic diversity and the screening of resistant materials is still undefined. Therefore, the purposes of this study were to: (i) evaluate the intricate relationships between biological characteristics and pathogenicity of *U. virens* in Heilongjiang province; (ii) determine the genetic diversity of the *U. virens*; and (iii) identify the resistant rice varieties of *U. virens* in Heilongjiang province.

## 2. Materials and Methods

### 2.1. Sample Collection and Isolation

From 2018 to 2023, 110 rice samples infected with rice false smut were collected in eight major cities of Heilongjiang province: the main rice-growing area, namely Harbin, Qiqihar, Jiamusi, Mudanjiang, Yichun, Jixi, Daqing and Suihua. The details of sampling from each site are presented in Supplementary Figure S1 and Supplementary Table S1. The rice ball was disinfected by soaking it in 70% alcohol for 1 minute, followed by rinsing the surface three times with sterile water. The surface water was dried with filter paper. The rice curd balls were cut into 4 pieces with a scalpel and cultured in PSA (Potato 200 g; Sucrose 20 g; agar 20 g; Water 1000 mL) solid medium for 5 days at 28 °C in the dark [22].

### 2.2. Pathogenicity Test

The white mycelium produced by the germination of yellow fungal chlamydospores was selected and cultured in PSA medium. After culturing on PSA medium for 7 days, 5 mm mycelium disks were placed into 150 mL flasks containing 100 mL potato sucrose (PS, made from a boiled extract of 200 g of peeled potatoes and 20 g of sucrose) fluid medium. The cultures were incubated at 28 °C on a shaker at 150 rpm for 7 days. The hyphae and conidia were collected by filtration and centrifugation, respectively. The mycelia were re-suspended in the PS and pulverized for five minutes with a crusher. The conidia were added back to the hyphae fragment suspension to produce a mixture of spores and hyphae fragments used for inoculation. After that, the liquid culture was used to make the mixture of conidia. The concentration of conidia was adjusted to 1 × 10^5^ conidia/mL, 24 μL spore solution was diluted with 96 μL sterile water and then coated with PSA agar for 2 days. For pathogenicity, the seeds were soaked in 10 mL of conidia suspension (1 × 10^5^ conidia/mL).

### 2.3. Morphological Characterization

All isolates were inoculated on PSA plates and incubated at 28 °C in the dark for 7 days. Each isolate was assessed based on colony color. In addition, these isolates were cultured on PSA at 28 °C for 7 days with a light/dark cycle of 8/16 h to observe the well-developed macroconidia using light microscopy (The Zeiss Axiolab 5 equipped with an Axiocam 208 color industrial digital camera is manufactured by Carl Zeiss in Oberkochen, Germany) [19]. A mycelium disk (5 mm) was made at the edge of the colony and placed in PS liquid for oscillating culture for 7 days to observe the color of the liquid culture. At the same time, 24 μL spore solution was diluted with 96 μL sterile water and coated on PSA plate medium [19,22]. After 48 h of culture under dark conditions, the morphology, color and mycelium germination and sporulation were observed under a microscope.

For the determination of hyphal growth rate, mycelium disks with a diameter of 5 mm were cultured on PSA solid medium, and each group was repeated 3 times. After dark culture at 28 °C for 7 days, the colony diameter was measured by the cross method [23,24]. According to the growth rate of mycelium, the growth rate of mycelium was divided into three types: fast mycelium growth rate (>3.0 mm/d), moderate (1.5~3 mm/d) and slow (<1.5 mm/d). Likewise, the sporulation ability was determined as follows: *U. virens* discs with a diameter of 5 mm were cultured in PSA solid medium under dark conditions at 28 °C for 7 days, and three discs with a diameter of 5 mm were placed in PS liquid medium at the edge of the colony at 150 rpm/min and oscillated at 28 °C for 7 days [23,24]. The sporulation was measured by hemocytometer counting plate. According to the sporulation capacity, the sporogenic capacity of *U. virens* was divided into 3 types: strong sporulation capacity (sporulation capacity > 250 × 10^4^ cells/mL), medium sporulation capacity (50~250 × 10^4^ spores/mL) and weak sporulation capacity (<50 × 10^4^ spores/mL).

Furthermore, the PSA plate spore germination method was used to determine the spore germination of *U. virens*. Conidia were coated on PSA solid medium; each group was coated with 3 replicates with dark culture at 28 ℃ for 48 h [5,23]. A total of 300 spores were detected in 3 agar blocks in each group, and the number of germination spores (spores with bud tube length greater than spore diameter) was recorded. According to the spore germination rate, *U. virens* was classified into three types: high spore germination rate (75%–100%), medium spore germination rate (35%–75%) and low spore germination rate (1–35%) [19,25]. The liquid culture color was identified as follows: the 5 mm diameter *U. virens* dish was placed in PSA solid medium and incubated at 28 ℃ for 7 days, 3 petri dishes with a diameter of 5 mm were placed in PS liquid medium at the edge of the colony at 150 rpm/min and the liquid color of the liquid culture was observed after 7 days of shaking culture at 28 ℃. White, yellow-green, dark green and dark yellow colors were noted.

### 2.4. Identification of Rice Disease Resistance

Our study used 86 *U. virens* strains isolated and purified from single spores. The tested rice varieties are Wuyoudao No. 4 (RFS disease susceptible variety), provided by the Qiqihar branch of the Heilongjiang Academy of Agricultural Sciences. All the selected isolates for the pathogenicity test were inoculated into PS liquid medium and cultured for 7 days on a rotary shaker at 150 rpm/min and 28 °C. The conidia suspension was filtered and adjusted to 1 × 10^5^ conidia/mL. Healthy rice plants (Wuyoudao No. 4) were grown in the greenhouse at the heading stage and were selected for the pathogenicity test. The flat leaf pillow or 1–4 cm apart (about 7 days before the break stage) was wounded with a sterile needle (1 mm diameter), and then, 10 µL of conidia suspension was injected into the rice sheaths and marked. As a control, three rice leaf sheaths were inoculated with 10 µL of sterile water. Symptoms were observed after 25 days post inoculation. According to the average number of diseased grains per spike, the pathogenicity of *U. virens* was divided into three types: strong pathogenicity (average number of diseased grains per spike > 26.4), moderate pathogenicity (average number of diseased grains per spike was 2.64–26.4) and weak pathogenicity (average number of diseased grains per spike < 2.64) [19,23,24,25].

### 2.5. Identification and Evaluation of Tolerance to RFS Disease

The 40 rice varieties tested in Heilongjiang province were provided by the Plant Pathology Laboratory of Northeast Agricultural University. In Wuchang City, Heilongjiang province, plots with false smut disease were selected. Rice seedlings were cultivated in spring, and field transplanting was carried out at the 3-leaf stage. Each variety was planted in 3 rows, each 3 meters long, and the experiment was repeated three times. After transplanting, conventional field water and fertilizer management and disease and pest control were carried out.

The quantitative indicators of the biological characteristics (mycelium growth rate, porulation, spore germination rate and liquid culture color) of *U. virens* strains were calculated. These indicators were used to evaluate the potential relationship between the pathogen’s characteristics and the rice varieties’ tolerance to RFS disease. Then, cluster analysis was performed using the sum of squares of deviations method to construct the cluster analysis diagram of *U. virens’* biological characteristics. This analysis aimed to assist in identifying groups of *U. virens* strains with similar characteristics and potentially correlating them with the tolerance levels of different rice varieties, providing a basis for evaluating the tolerance of rice varieties to RFS disease.

### 2.6. Molecular Identification and Genetic Diversity Analysis

The genomic DNA of the strains were extracted using a Fungal Genomic DNA Rapid Extraction Kit (Solarbio, Beijing, China). The DNA strains of *U. virens* were identified by PCR using specific primers US1-5:5′-CCGGAGGATACAACCAAAAAAACTCT-3′andUS3-3:5′-GCTCCAAGTGCGAGGATAACTGAAT-3 [24]. The PCR amplification procedure was as follows: pre-denaturation at 94 ℃ for 3 min; denatured at 94 ℃ for 30 s, annealed at 58 ℃ for 30 s, extended at 72 ℃ for 1 min, 30 cycles; extended at 72 ℃ for 7 min. The DNA of *U. virens* strains was identified using specific primers of BOX-PCR, ERIC-PCR and REP-PCR. The forward 5′-CTACGGCAAGGCGACGCTGACG-3′/5′-ATGTAAGCTCCTGGGGATTCAC-3′/IIIICGICGI-CATCIGGC-3′andreverseprimers5′-CTACGGCAAGGCGACGCTGACG-3′/5′-AAGTAAGTGACTGGGGTGAGCG-3′/5′-ICGICTTATCIGGCCTAC-3′ of BOX-PCR, ERIC-PCR and REP-PCR were used to amplify the partial DNA from 86 strains of *U. virens* [22,26]. The 5 μL amplified product was electrophoresed on a 2% agarose gel in 1 × TAE buffer at 120 V for 80 min and photographed by the gel imaging system.

Each band in the electrophoretic spectrum was used as a molecular marker, and the value was assigned as “1” at the band and “0” at the non-band. The “0, 1” data matrix was established for the next step of data analysis. The above data were processed by NTSYS-pc (version 2.1), and the tree cluster analysis graph was established by UPGMA method. PopGen 32 software was used to calculate population genetic diversity, genetic distance and genetic consistency. Molecular analysis of Variance (AMOVA) was conducted to calculate the inter–population genetic differentiation coefficient.

### 2.7. Data Analysis

Two-dimensional scatter plots of mycelium growth rate, sporulation amount, spore germination rate, liquid culture color and pathogenicity were analyzed using SPSS (v. 20.0; SPSS Inc., Wacker Drive, Chicago, IL, USA. IBM Corp., Armonk, NY, USA, 2012. IBM), and the correlation coefficient R value was calculated. According to the size of R value, the correlations were divided into the following types: R < 0 was negative correlation; R = 0 had no linear correlation; |R| > 0.95 was a significant correlation. Moreover, 0.5 ≤ |R| < 0.8 was moderately correlated; 0.3 ≤ |R| < 0.5 was lowly correlated; |R| < 0.3 was a very weak relationship. A difference was considered significant at *p* ≤ 0.05. Origin software (OriginLab Corporation, v.9.9.0.225 (SR1) Northampton, MA, USA, 2022) was used to perform principal component analysis (PCA) of the correlation between pathogenicity and spore production, germination rate and liquid culture pigments.

## 3. Results

### 3.1. Morphological Identification

**Figure 1:**
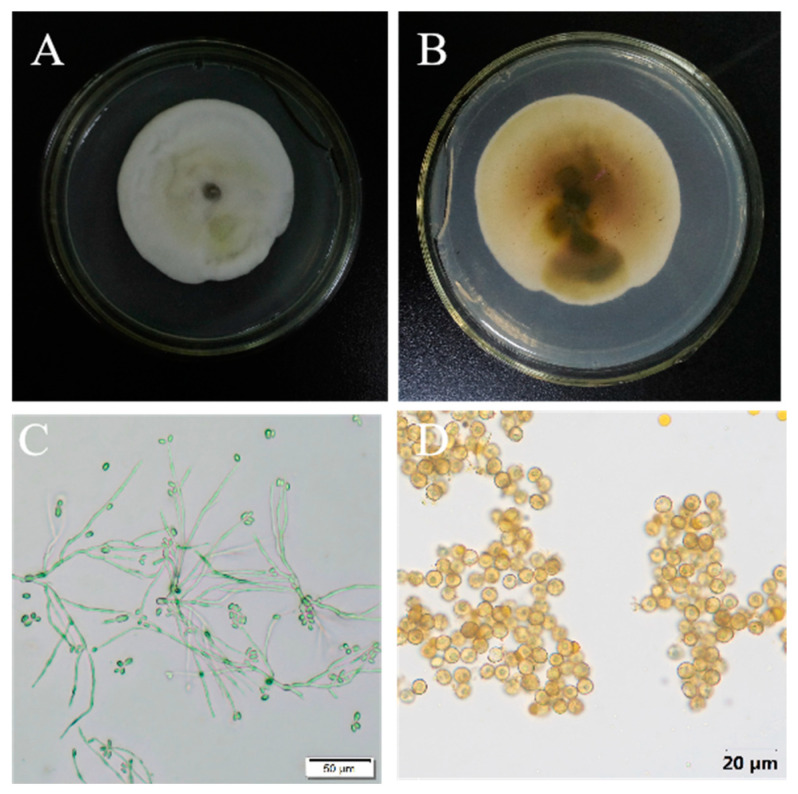
Morphological identification of *Ustilaginoidea virens*. Note: (**A**) colony morphology; (**B**) color of the back of the colony; (**C**) thin-wall conidia; (**D**) chlamychospore.

Morphological characteristics (mycelium growth rate, sporulation, spore germination rate and liquid culture color) and molecular biology analysis identified a total of 86 strains, distributed as follows: 15 in Harbin, 13 in Qiqihar, 11 in Jiamusi, 13 in Mudanjiang, 7 in Yichun, 11 in Jixi, 9 in Daqing and 7 in Suihua. The mycelium appeared white and dense after 7 days of colony culture. Following a 14-day colony culture period, the colony color progressively changed from yellow to yellowish brown with irregular colony edges, and the middle of the colony was convex (Figure 1A). The color of the colony back gradually changed from white to yellow, yellow-green or dark green later (Figure 1B). The translucent, round or elliptic, colorless conidium spore had a diameter of around 2–10 μm. Mycelium was created when thin-walled conidia germinated. On the mycelium, secondary conidia first appeared, followed by tertiary conidia (Figure 1C). The majority of chlamydospores had an oval or circular, pale yellow, yellow-green or black structure (Figure 1D).

### 3.2. Correlation Between Biological Characteristics and Pathogenicity

**Figure 2:**
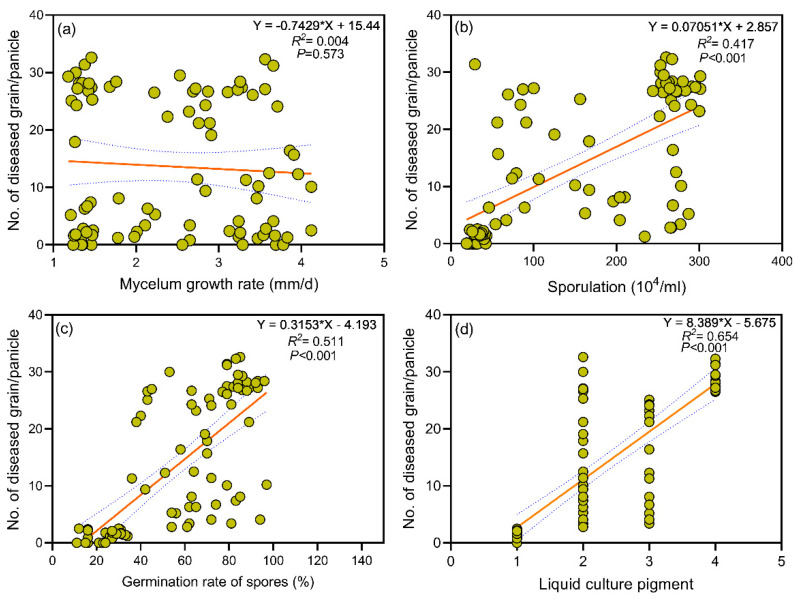
The scatter plot shows the relationship between (**a**) the mycelium growth rate of *Ustilaginoidea virens* and the number of disease grains; (**b**) the spore production quantity of *Ustilaginoidea virens* and the number of disease grains; (**c**) the spore germination rate of *Ustilaginoidea virens* and the number of disease grains; (**d**) the liquid culture color of *Ustilaginoidea virens* and the number of disease grains. Note: In (**d**), 1–4 represent white, yellow-green, dark green and dark yellowish green culture colors, respectively.

The biological characteristics of 86 strains of RFS disease are presented in Table 1. A high growth rate of mycelium often implies a fast diffusion speed within the host. The generation of a large number of spores increases the chance of the pathogen spreading to new hosts. A high spore germination rate is usually conducive to the rapid establishment of infection by the pathogen. The color of liquid culture sometimes can reflect the metabolites of the pathogen. Some metabolites may be related to the pathogenicity. Therefore, we analyzed the correlations between the morphology (mycelium growth rate, sporulation, germination rate of spores, liquid culture color) and pathogenicity. The relationships between the mycelium growth rate and pathogenicity were not significant (*p* = 0.58 and R = 0.004), and no correlation was observed (|R| < 0.3) (Figure 2a). Among the 86 strains isolated in Heilongjiang province, the numbers of fast, medium and slow mycelium growth rates were 31, 27 and 28, respectively. Similarly, with the significant probability of *p* = 0.001 and correlation coefficient R = 0.41, the sporulation ability of *U. virens* was positively correlated with pathogenicity (0.5 ≤ |R| < 0.8) (Figure 2b). The numbers of strains with high, medium and weak sporulation ability were 33, 27 and 26 respectively, in Heilongjiang province. There was a strong positive correlation between spore germination and pathogenicity (*p* = 0.001 and R = 0.51; |R| > 0.8) (Figure 2c). There were 29, 30 and 27 strains with strong, medium and weak spore germination respectively, in Heilongjiang province. The study found a considerable positive correlation between liquid culture color and pathogenicity, with statistical significance (*p* = 0.001, R = 0.65) (Figure 2d). The number of white, yellowish green, dark green and dark yellow strains were 27, 24, 17 and 18, respectively (Supplementary Figure S2). The pathogenicity of a strain increased with increased sporulation, spore germination capabilities and dark color in the liquid culture. The pathogenicity test of the RFS pathogen is illustrated in Supplemental Figure S2. PCA indicated that the liquid culture color was the main factor affecting pathogenicity (Supplemental Figure S3).

### 3.3. Analysis of the Clustering Situation of U. virens in Heilongjiang Province

**Figure 3:**
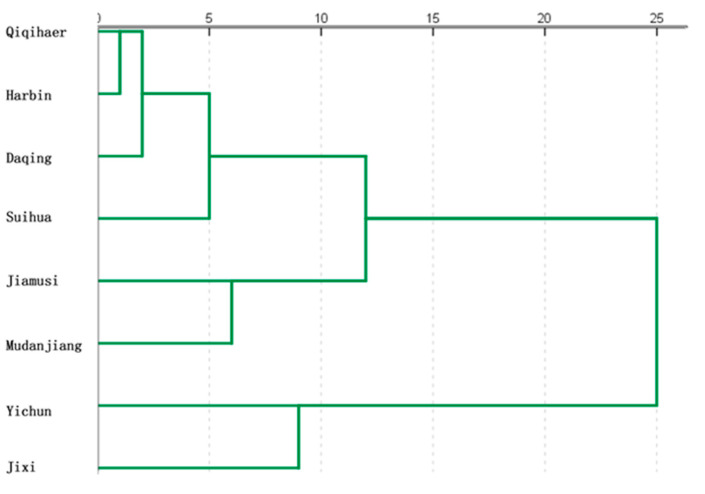
Dendrogram based on biological characteristics of *Ustilaginoidea virens* in Heilongjiang province. The branch length represents the distance or degree of difference between clusters. The shorter the length was, the higher the similarity between the corresponding clusters was, and vice versa.

The biological characteristics and frequency of RFS disease in different regions of the province are presented in Supplementary Table S1. The square Euclidean distance between *U. virens* strains in Heilongjiang province was computed based on 18 quantitative indicators of biological features, and cluster analysis was performed using the deviation square sum method to construct a cluster analysis diagram of biological characteristics of *U. virens* strains (Figure 3). The findings revealed that when the square Euclidean distance was 10, the biological properties of *U. virens* strains in Heilongjiang province were classified into three groups. Qiqihar, Harbin, Daqing and Suihua were in Category 1; Jiamusi and Mudanjiang were in Category 2; while Yichun and Jixi were in Category 3. Cluster analysis revealed that each region disease strains had distinct biological properties. The biological properties of *U. virens* strains in neighboring locations were comparable, and they were classified as one type on the cluster analysis tree, such as Qiqihar and Harbin, Daqing and Suihua, and Jiamusi and Mudanjiang. In the cluster analysis tree, the *U. virens* strains from far-off places like Harbin, Jixi and Yichun were not clustered together because of their significantly dissimilar biological traits.

### 3.4. Rep-PCR Primers Amplification

The DNA fingerprints of isolates from *U. virens* amplified by primer ERIC-PCR contained 5–12 major strands, with fragment sizes ranging from 250 to 5000 bp and a genetic diversity value of 0.79 (Supplementary Figure S4A). The fingerprint amplified by primer BOX-PCR had 8–14 major strands, fragment sizes ranging from 200 to 3000 bp and a genetic diversity score of 0.87 (Supplementary Figure S4B). The fingerprint amplified using primer REP-PCR had 5–10 major fragments with a fragment size of 250–5000 bp and a genetic diversity value of 0.74 (Supplementary Figure S4C). All three primers had genetic diversity values larger than 0.7, making them suitable for cluster analysis. However, BOX-PCR primers had the maximum number of primary bands, the greatest genetic diversity and the best band polymorphism.

### 3.5. Cluster Analysis of U. virens

**Figure 4:**
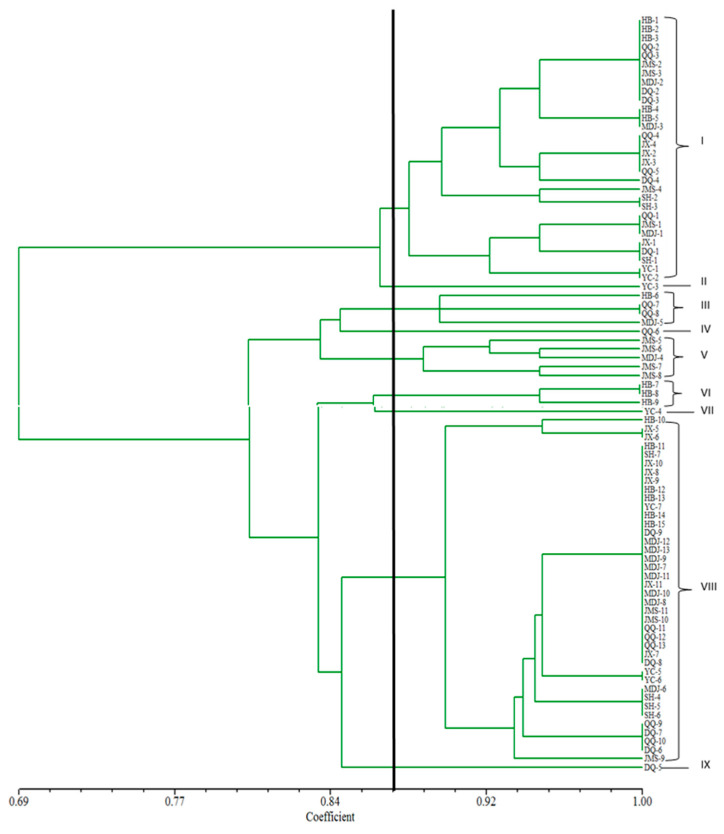
Dendrogram of 86 *Ustilaginoidea virens* strains in Heilongjiang province based on the molecular fingerprinting patterns by BOX-PCR.

The cluster analysis of 86 strains of rice false smut was performed (Figure 4). The 86 strains were categorized into nine gene groups based on their similarity coefficients ranging from 0.69 to 1, with 0.87 serving as the boundary. Group I had 30 strains, accounting for 34.9% of all strains tested. The strains were from Harbin, Qiqihar, Jiamusi, Mudanjiang, Daqing, Jixi, Suihua and Yichun. There were four strains in Group III, accounting for 4.7% of the total strains tested, and the strains were from Harbin, Qiqihar and Mudanjiang. There were five strains in Group V, accounting for 5.8% of the total strains tested, and the strains were from Jiamusi and Mudanjiang. There were three strains in Group VI, accounting for 3.5% of the total strains, all of which came from Harbin. There were 40 strains in Group VIII, accounting for 46.5% of the total strains, and the strains were from Harbin, Qiqihar, Jiamusi, Mudanjiang, Yichun, Jixi, Daqing and Suihua. Group II, IV, VII and IX had only one strain from Yichun, Qiqihar, Yichun and Daqing, respectively, accounting for 4.7% of the total strains tested. The results showed that the structure of *U. virens* was diverse and complex in Heilongjiang province. The results of genetic differentiation analysis showed that the maximum coefficient of genetic differentiation was 0.1786 between Harbin and Yichun, and the minimum coefficient of genetic differentiation was 0.0129 between Harbin and Suihua. The population differentiation had a significant correlation with pathogenicity of *U. virens* in Heilongjiang but no significant correlation with gene groups and geographical region. The variation of different gene populations was mainly within population, which covered 95.63%, and between populations, it covered 4.37% (*p* > 0.5). The variation of different geographical populations was mainly within population, which covered 95.79%, and between populations, it covered 4.21% (*p* > 0.5). The variation of different pathogenicity populations was mainly within the population, which covered 86.08%, and between populations, it covered 13.92% (*p* < 0.5). There was a moderate degree of variation among populations of different pathogenicity. The pathogenicity had a significant effect on the differentiation of *U. virens* in Heilongjiang province.

### 3.6. Analysis of Genetic Diversity in Geographical Population

The genetic diversity of the geographical population of *U. virens* in Heilongjiang province was analyzed using PopGen 32 analysis software. The total observed number of alleles and the effective number of alleles were 1.7646 and 1.5608, respectively (Table 2). The observed number of alleles and the effective number of alleles ranged from 1.4896 to 1.7423 and from 1.2942 to 1.5467 among eight geographical populations. The highest values of the two indexes in Harbin were 1.7423 and 1.5467, while the lowest values of the two indexes in Yichun were 1.4896 and 1.2942 (Table 2). The total Nei’s gene diversity index value of *U. virens* was 0.3998, and the Nei’s gene diversity index value of eight geographical populations ranged from 0.1121 to 0.3793, among which the highest value was 0.3793 in Harbin and the lowest value was 0.1121 in Yichun (Table 2).

The Shannon information index value of the total population was 0.4979, and the Shannon information index value of the eight geographical populations ranged from 0.2434 to 0.4769, among which the highest value was 0.4769 in Harbin and the lowest value was 0.2434 in Yichun (Table 2).

The percentage of polymorphic loci was 43.9%, and the percentage of polymorphic loci in eight geographic populations ranged from 17.5% to 40.4%, with the highest value in Harbin and the lowest value in Yichun (Table 2). The genetic richness of *U. virens* in Heilongjiang province was in the following order from high to low: Harbin > Qiqihar > Daqing > Mudanjiang > Suihua > Jiamusi > Jixi > Yichun.

### 3.7. Identification and Evaluation of Tolerance to RFS Disease

**Figure 5:**
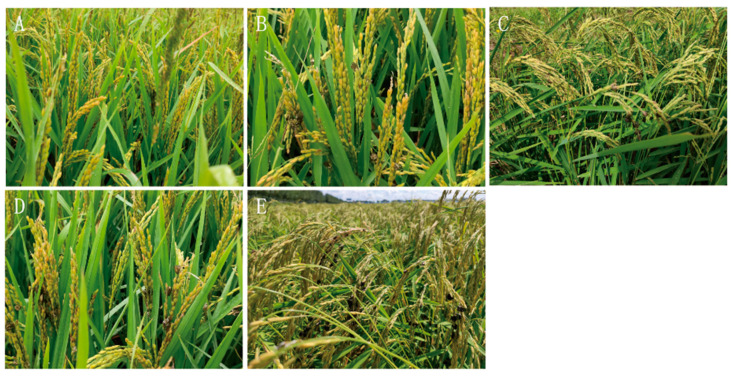
Tolerance grade of rice-to-rice false smut. Note: (**A**) tolerance, (**B**) moderately tolerant, (**C**) moderately susceptible, (**D**) susceptible, (**E**) highly susceptible. The figure shows a schematic diagram of the different resistances of field rice to the rice false smut.

The field natural incidence approach was used to identify the tolerance of 40 rice varieties to RFS disease in Heilongjiang province. The results showed that there were five types of tolerance: tolerance (R), moderate tolerance (MR), moderate susceptibility (MS), susceptible (S) and high susceptibility (HS) among the 40 rice varieties, and there were no immune varieties to RFS disease (Figure 5). Table 3 shows the tolerance identification results of 40 main rice varieties to RFS disease. The resistant varieties included Suijing 15, Longjing 60 and Mudanjiang 35, accounting for 7.5% of the total number. There were eight varieties with moderate tolerance, including Suijing 14, Songjing Xiang 2 and Kendao 12, accounting for 20% of the total (Table 3). There were nine middle-sensing varieties, including Longdao 21, Longjing 51, Longjing 29 and Longjing Xiang 1, accounting for 22.5% of the total. The susceptible rice varieties were Songjing 19, Songjing 9 and Longjing 61, which accounted for 20% of the total rice varieties. There were 12 rice varieties with high susceptibility, including Longjing 25, Youdao4 and Songmucui 1, accounting for 30% of the total (Table 3). On the whole, the ratio of resistant varieties to sensitive varieties was about 5:13, and the number of highly sensitive varieties accounted for 30% of the total (Table 3). Collectively, the results indicate that 3 rice varieties were resistant, 8 rice varieties were moderately resistant, 9 were moderately susceptible, 8 were susceptible and 12 were highly susceptible. The tolerance of rice varieties to *U. virens* in Heilongjiang province was poor, and most of rice varieties were susceptible to the disease.

## 4. Discussion

In this study, the RFS pathogen strains in Heilongjiang province were identified. The results of morphological identification and molecular identification were consistent, which significantly enhanced the reliability of the identification results. This means that we can be more confident that the identity of the identified species is accurate, reducing the misjudgment caused by the potential errors or limitations of a single method. Furthermore, the biological characteristics of 86 RFS pathogen strains were evaluated, and it was found that there were significant differences in mycelium growth rate, sporulation ability, spore germination and liquid culture color among different strains. These findings are compatible with those of [5,22,23]. Likewise, by measuring radial mycelial growth at 10-day intervals, we observed the pathogen development, including colony color, colony diameter, growth type and mycelia dry weight, after 30 days. It was found that spore germination ability and liquid culture color were positively correlated with pathogenicity. The study found that spore germination and liquid culture color significantly impact the pathogenicity of *U. virens*, with germination having a greater impact than sporulation ability. This discovery has significant implications for the research of *U. virens* pathogenicity. The synergistic effect of sporulation ability and spore germination on the pathogenicity of *U. virens* remains to be further studied.

The biological characteristics of the strains in close geographical locations were relatively similar, and they were often grouped together in the cluster analysis tree, such as Qiqihar and Harbin. The biological characteristics of strains of RFS in geographically distant regions were significantly different, and the distance in the cluster analysis tree diagram was relatively large, such as Harbin and Jixi. There was no obvious division of biological characteristics of strains of the disease in different regions indicating that different geographical origin had no obvious division of virulence. A recent study demonstrated that sclerotia may occur in a range of geographical places [26]. After a 2- to 5-month period of dormancy, sclerotia germinate and generate ascospores in the presence of light. Ascospores are commonly found in rice paddy fields before and after planting, demonstrating that sclerotia can overwinter effectively and routinely generate ascospores. Chlamydospores were first detected in rice paddy areas after disease symptoms began to appear. Infected grains were twice the diameter of normal grains. These were first yellow and then turned black [27]. In the early stages of infection, only a few grains were infected, but in severe infection, the entire field was contaminated [7]. This pathogen produced copious quantities of mycotoxins, including *Ustilaginoidins* and *Ustiloxins* [28]. Toxins inhibited rice plant development at both the shoot and root levels [29]. Ustiloxins are cyclic peptide derivatives that are toxic to plants and animals. Ustiloxins primarily inhibit the cytoskeleton development and microtubule assembly [22]. In a recent investigation, ustiloxins A and B were discovered in varied amounts in 240 samples of rice from China and 72 samples from 12 other countries [30]. Rice contamination in various regions is a significant issue involving the potential health hazards of ustiloxins, which are currently under investigation for potential RFS harm.

RFS, caused by *U. virens*, is a rare fungal disease with limited scientific data, particularly regarding population structure and genetic variation. In this study, Rep-PCR was employed to analyze genetic diversity. The results indicated high DNA band polymorphism and complex genetic diversity. The division of gene groups showed no obvious correlation with geographical origin. There was no significant correlation between gene groups, biological characteristics and geographical origin. Similar to previous research, Tan et al. found that most *U. virens* strains from various parts of Hunan province were genetically identical [31]. Higher genetic similarity among isolates from different fields suggests that the selection of *U. virens* isolates is more influenced by geographic variables. Similarly, for 110 strains of *U. virens* collected from 10 rice-producing areas in China, there was no correlation between gene groups and biological characteristics, but the division of gene groups was significantly affected by geographical origin [31,32,33,34,35]. The geographical location had a significant impact on the genetic diversity of *U. virens* compared to rice cultivars [35,36]. The first findings on genetic variation and population structure of *U. virens* isolates from India revealed that the broad regions of eastern and northeastern India produced a large number of isolates, some from distinct rice fields, increasing the variety. According to the study, all *U. virens* populations had low genetic diversity, with a similarity of more than 60%, and some influenced from location. A comprehensive understanding of *U. virens* variety and population structure is crucial for RFS disease management measures and the use of disease-tolerant rice lines.

To date, the correlation between the genetic variation of *U. virens* and geographical environment has been widely studied and discussed with some controversy. Yang et al. [35] pointed out that the clustering of isolates had no clear relationship with their geographical location. However, Fang et al. [36] found a high degree of genetic variation of *U. virens* among geographical populations. The results of this study showed that most populations showed significant genetic differentiation. This is consistent with the conclusion that geographical environment has a vital influence on the fungal population. The genetic consistency and genetic distance of geographically close populations tended to be higher; for example, the genetic consistency of Harbin and Qiqihar was 0.9902. The reason may have been the close geographical location, similar climatic conditions and gene exchange between the two populations, which made the two populations have high genetic consistency. The minimum genetic consistency between Jixi and Daqing was 0.9299, which may have been due to the geographical distance between Jixi and Daqing, and the lack of gene exchange between the two communities, thus forming a relatively independent gene population structure. On the microcosmic level, host–pathogen interaction and variety resistance resulted in variations in pathogen virulence, sexual reproduction, gene mutation and recombination, chromosomal variation and so on. On a macro level, meteorological circumstances, geographical environment, farming system and cultivation conditions may have all influenced *U. virens* variation. Other explanations for *U. virens’* great genetic diversity require further investigation.

## 5. Conclusions

This study evaluated the biological characteristics, pathogenicity and genetic diversity of *U. virens* in Heilongjiang province, as well as the identification of rice varieties resistant to *U. virens*. The study found diverse mycelium growth rates, sporulation abilities and spore germination levels among the strains. The population structure of *U. virens* in Heilongjiang province was diverse and complex. The genetic diversity of the geographic population varied. There was also genetic differentiation among different regions. Molecular variance analysis showed that the variation of different gene groups was mainly within the population. The disease resistance of varieties was evaluated, and it was concluded that the resistance of rice varieties to RFS in Heilongjiang province was generally poor, and most were susceptible. In conclusion, strengthening the monitoring of genetic variation and analysis of structural genetic diversity of *U. virens* in various regions is of great significance for the prevention and control of RFS and breeding resistant varieties.

## Figures and Tables

**Table 1:** The determination results of the biological characteristics of *Ustilaginoidea virens* in Heilongjiang province.

Strain Number	Region	Mycelial Growth Rate (mm/d)	Sporulation (10^4^/mL)	Germination Rate of Spores (%)	Liquid Culture Color	No. of Diseased Grain
B-1	Harbin	3.56 ± 0.12	301 ± 20.06	89 ± 8.93	YG	27.1 ± 2.11
B-2	Harbin	1.78 ± 0.21	234 ± 17.87	34 ± 3.23	W	1.2 ± 0.21
HB-3	Harbin	1.21 ± 0.34	287 ± 18.76	56 ± 4.56	DG	5.2 ± 2.12
HB-4	Harbin	3.13 ± 0.07	23 ± 9.67	16 ± 1.34	W	2.4 ± 1.01
HB-5	Harbin	1.22 ± 0.13	266 ± 31.45	43 ± 9.12	DG	25.1 ± 3.21
HB-6	Harbin	3.69 ± 0.03	28 ± 5.34	11 ± 4.56	W	0 ± 0
HB-7	Harbin	1.68 ± 0.06	291 ± 32.11	79 ± 11.23	DY	27.5 ± 3.21
HB-8	Harbin	2.64 ± 0.09	300 ± 16.67	65 ± 13.45	DG	23.2 ± 2.34
HB-9	Harbin	1.46 ± 0.11	26 ± 6.67	16 ± 4.56	W	0 ± 0
HB-10	Harbin	3.28 ± 0.23	265 ± 12.34	82 ± 8.69	DY	27.4 ± 6.12
HB-11	Harbin	1.31 ± 0.06	254 ± 9.08	84 ± 7.44	DY	28.1 ± 3.45
HB-12	Harbin	3.48 ± 0.08	150 ± 11.34	97 ± 3.54	YG	10.2 ± 2.34
HB-13	Harbin	2.65 ± 0.05	277 ± 20.03	62 ± 2.56	YG	3.4 ± 1.22
HB-14	Harbin	2.23 ± 0.14	162 ± 8.97	54 ± 2.21	YG	5.3 ± 1.12
HB-15	Harbin	3.61 ± 0.25	272 ± 13.45	64 ± 2.11	YG	12.5 ± 4.56
QQ-1	Qiqihar	1.27 ± 0.18	253 ± 21.33	53 ± 1.98	YG	30.1 ± 5.12
QQ-2	Qiqihar	2.22 ± 0.17	256 ± 17.89	77 ± 7.02	DY	26.5 ± 2.11
QQ-3	Qiqihar	3.66 ± 0.08	67 ± 4.32	72 ± 6.97	YG	4.1 ± 1.12
QQ-4	Qiqihar	3.78 ± 0.19	22 ± 3.21	21 ± 2.32	W	0 ± 0
QQ-5	Qiqihar	4.12 ± 0.22	278 ± 16.76	79 ± 6.77	YG	10.1 ± 1.32
QQ-6	Qiqihar	3.11 ± 0.08	273 ± 15.32	43 ± 3.21	YG	26.6 ± 2.34
QQ-7	Qiqihar	1.35 ± 0.16	30 ± 5.43	11 ± 2.34	W	0 ± 0
QQ-8	Qiqihar	2.89 ± 0.17	56 ± 6.32	89 ± 7.89	YG	21.2 ± 3.23
QQ-9	Qiqihar	2.38 ± 0.2	252 ± 21.44	40 ± 2.33	DG	22.3 ± 2.34
QQ-10	Qiqihar	2.11 ± 0.21	54 ± 9.02	81 ± 5.43	DG	3.4 ± 0.41
QQ-11	Qiqihar	2.01 ± 0.25	35 ± 8.77	31 ± 2.34	W	2.3 ± 0.22
QQ-12	Qiqihar	1.26 ± 0.27	167 ± 12.34	70 ± 6.43	YG	17.9 ± 3.21
QQ-13	Qiqihar	1.33 ± 0.15	32 ± 3.45	16 ± 4.56	W	0.9 ± 0.23
JMS-1	Jiamusi	1.38 ± 0.06	29 ± 2.34	79 ± 3.23	DY	31.4 ± 9.23
JMS-2	Jiamusi	1.34 ± 0.16	32 ± 3.12	23 ± 2.12	W	0 ± 0.
JMS-3	Jiamusi	1.46 ± 0.17	295 ± 23.12	82 ± 4.56	DY	27.4 ± 3.21
JMS-4	Jiamusi	1.98 ± 0.24	43 ± 4.65	32 ± 2.12	W	1.4 ± 0.11
JMS-5	Jiamusi	3.24 ± 0.07	204 ± 3.42	94 ± 5.67	DG	4.1 ± 1.16
JMS-6	Jiamusi	3.46 ± 0.08	210 ± 14.56	63 ± 3.23	DG	8.1 ± 3.22
JMS-7	Jiamusi	1.42 ± 0.26	283 ± 16.54	86 ± 4.34	DY	26.8 ± 7.21
JMS-8	Jiamusi	1.38 ± 0.28	89 ± 6.78	62 ± 2.45	YG	6.3 ± 1.23
JMS-9	Jiamusi	1.36 ± 0.15	266 ± 18.91	61 ± 3.02	YG	2.8 ± 1.45
JMS-10	Jiamusi	3.83 ± 0.24	40 ± 4.23	28 ± 2.12	W	1.3 ± 0.23
JMS-11	Jiamusi	1.28 ± 0.13	290 ± 23.12	81 ± 7.21	DG	24.3 ± 2.34
MDJ-1	Mudanjiang	2.14 ± 0.21	46 ± 4.32	65 ± 2.12	YG	6.3 ± 1.09
MDJ-2	Mudanjiang	3.23 ± 0.06	87 ± 2.12	45 ± 3.21	YG	27 ± 4.56
MDJ-3	Mudanjiang	1.26 ± 0.23	32 ± 1.32	24 ± 2.12	W	1.8 ± 0.18
MDJ-4	Mudanjiang	1.45 ± 0.18	196 ± 12.43	83 ± 6.21	YG	7.4 ± 2.23
MDJ-5	Mudanjiang	1.76 ± 0.16	267 ± 14.22	93 ± 4.54	DY	28.4 ± 3.45
MDJ-6	Mudanjiang	3.47 ± 0.23	38 ± 9.32	26 ± 3.45	W	1.1 ± 0.22
MDJ-7	Mudanjiang	2.91 ± 0.17	125 ± 3.02	69 ± 5.61	YG	19.1 ± 2.34
MDJ-8	Mudanjiang	1.24 ± 0.07	42 ± 2.12	16 ± 2.21	W	0 ± 0
MDJ-9	Mudanjiang	2.65 ± 0.11	40 ± 2.32	27 ± 1.23	W	0.8 ± 0.34
MDJ-10	Mudanjiang	2.84 ± 0.24	167 ± 8.45	42 ± 3.21	YG	9.4 ± 2.33
MDJ-11	Mudanjiang	3.86 ± 0.08	268 ± 10.22	58 ± 2.34	DG	16.4 ± 4.34
MDJ-12	Mudanjiang	1.35 ± 0.01	263 ± 9.32	88 ± 5.67	DY	28.2 ± 6.11
MDJ-13	Mudanjiang	1.47 ± 0.12	156 ± 4.22	71 ± 4.53	YG	25.3 ± 3.21
YC-1	Yichun	1.46 ± 0.11	260 ± 5.67	85 ± 5.65	YG	32.6 ± 4.23
YC-2	Yichun	2.56 ± 0.03	19 ± 2.11	24 ± 4.57	W	0 ± 0
YC-3	Yichun	3.96 ± 0.3	79 ± 4.23	51 ± 3.24	DG	12.3 ± 3.22
YC-4	Yichun	3.24 ± 0.28	35 ± 2.13	33 ± 2.14	W	1.4 ± 0.19
YC-5	Yichun	2.69 ± 0.19	272 ± 10.05	63 ± 3.42	YG	26.7 ± 2.34
YC-6	Yichun	1.79 ± 0.14	204 ± 9.88	85 ± 5.62	YG	8.1 ± 3.22
YC-7	Yichun	1.48 ± 0.17	32 ± 1.23	30 ± 2.11	W	2.5 ± 1.21
JX-1	Jixi	1.47 ± 0.07	41 ± 2.34	31 ± 2.31	W	1.7 ± 0.67
JX-2	Jixi	2.84 ± 0.18	84 ± 4.56	63 ± 3.21	DG	24.3 ± 5.67
JX-3	Jixi	2.74 ± 0.19	74 ± 5.61	72 ± 2.43	YG	11.4 ± 3.34
JX-4	Jixi	2.87 ± 0.22	244 ± 13.22	88 ± 3.22	DY	26.7 ± 2.14
JX-5	Jixi	3.91 ± 0.27	57 ± 3.23	70 ± 2.22	YG	15.7 ± 2.23
JX-6	Jixi	1.25 ± 0.07	38 ± 10.12	31 ± 1.47	W	1.6 ± 0.08
JX-7	Jixi	3.26 ± 0.23	280 ± 20.34	96 ± 7.32	DY	28.4 ± 2.12
JX-8	Jixi	2.72 ± 0.19	100 ± 3.44	93 ± 6.72	DY	27.2 ± 3.21
JX-9	Jixi	3.33 ± 0.12	106 ± 5.65	36 ± 4.35	DG	11.3 ± 2.11
JX-10	Jixi	1.4 ± 0.05	39 ± 2	16 ± 2.33	W	2.2 ± 0.23
JX-11	Jixi	1.18 ± 0.02	301 ± 17.21	86 ± 7.45	DY	29.3 ± 4.22
DQ-1	Daqing	3.56 ± 0.18	267 ± 16.71	83 ± 2.66	DY	32.3 ± 4.67
DQ-2	Daqing	2.76 ± 0.15	91 ± 5.21	38 ± 2.11	DG	21.2 ± 4.56
DQ-3	Daqing	4.12 ± 0.31	32 ± 12.2	12 ± 1.22	W	2.5 ± 0.22
DQ-4	Daqing	1.43 ± 0.07	260 ± 15.21	92 ± 9.23	DY	28.1 ± 3.12
DQ-5	Daqing	3.54 ± 0.12	28 ± 4.5	24 ± 3.02	W	1.7 ± 0.11
DQ-6	Daqing	3.66 ± 0.16	253 ± 11.21	79 ± 4.22	DY	31.2 ± 2.11
DQ-7	Daqing	2.53 ± 0.18	257 ± 12.23	84 ± 2.31	DY	29.5 ± 3.21
DQ-8	Daqing	3.29 ± 0.19	22 ± 5.23	15 ± 1.13	W	0 ± 0
DQ-9	Daqing	3.71 ± 0.26	270 ± 13.45	72 ± 5.23	DG	24.1 ± 2.33
SH-1	Suihua	1.27 ± 0.03	37 ± 3.12	27 ± 2.33	W	1.8 ± 0.12
SH-2	Suihua	3.68 ± 0.24	35 ± 2.12	29 ± 3.21	W	1.6 ± 0.22
SH-3	Suihua	3.57 ± 0.21	265 ± 14.23	54 ± 4.56	YG	2.8 ± 0.67
SH-4	Suihua	1.41 ± 0.04	268 ± 15.67	74 ± 6.44	DG	6.7 ± 1.21
SH-5	Suihua	1.29 ± 0.05	264 ± 24.23	84 ± 7.11	DY	27.2 ± 3.24
SH-6	Suihua	3.41 ± 0.26	69 ± 6.34	79 ± 6.43	YG	26.1 ± 4.23
SH-7	Suihua	3.26 ± 0.17	26 ± 3.23	27 ± 2.33	W	2.1 ± 0.78

**Table 2:** Genetic diversity analysis of geographic population of *Ustilaginoidea virens* in Heilongjiang province.

Region	No. of Samples	Observed Number of Alleles	Effective Number of Alleles	Nei’s Gene Diversity Index	Shannon Information Index	No. of Polymorphic Loci	No. of Polymorphic Loci (%)
Harbin	15	1.7423	1.5467	0.3793	0.4769	23	40.4%
Qiqihar	13	1.7264	1.5249	0.3576	0.4563	21	36.8%
Jiamusi	11	1.5869	1.3836	0.2394	0.3403	13	22.8%
Mudanjiang	13	1.6657	1.4694	0.3002	0.4069	17	29.8%
Yichun	7	1.4896	1.2942	0.1121	0.2434	10	17.5%
Jixi	11	1.5742	1.3724	0.2289	0.3376	11	19.3%
Daqing	9	1.6873	1.4869	0.3231	0.4279	18	31.6%
Suihua	7	1.6214	1.4268	0.2679	0.3763	15	26.3%
Average	11	1.6367	1.4381	0.2761	0.3832	16	28.1%
Total	86	1.7646	1.5608	0.3998	0.4979	25	43.9%

**Table 3:** Evaluation results of RFS tolerance of 40 rice varieties in Heilongjiang province.

Number	Rice Varieties	Average Disease Head Rate%	Classification of Disease Resistance	Number	Rice Varieties	Average Disease Head Rate%	Classification of Disease Resistance
1	Suijing 15	2.2	R	21	Pine japonica 19	17.2	S
2	Mudanjiang 35	2.2	R	22	Pine japonica 9	17.2	S
3	Dragon japonica 60	0.9	R	23	Dragon japonica 61	17.2	S
4	Suijing 14	4.2	MR	24	Dragon japonica 53	17.1	S
5	Dragon japonica 56	4.1	MR	25	Dragon japonica 47	17.2	S
6	Dragon japonica 55	3.4	MR	26	Dragon japonica 39	18.2	S
7	Dragon japonica 43	4.5	MR	27	Dragon japonica 21	17.1	S
8	Dragon japonica 31	4.1	MR	28	Long Yang 11	18.4	S
9	Pine japonica 2	4.2	MR	29	Dragon Rice 18	27.4	HS
10	Pine japonica 1	5.1	MR	30	Suijing 18	25.3	HS
11	Cultivated rice 12	6.1	MR	31	Pine japonica 22	25.4	HS
12	Dragon Rice 21	8.1	MS	32	Pine japonica 18	26.1	HS
13	Suijing 4	9.2	MS	33	Pine japonica 16	26.3	HS
14	Dragon japonica 58	11.2	MS	34	Dongnong 425	25.3	HS
15	Dragon japonica 54	9.6	MS	35	Dragon japonica 59	27.1	HS
16	Dragon japonica 51	7.4	MS	36	Dragon japonica 57	26.3	HS
17	Dragon japonica 50	10.7	MS	37	Dragon japonica 52	27.4	HS
18	Dragon japonica 46	8.5	MS	38	Dragon japonica 25	26.6	HS
19	Dragon japonica 29	8.2	MS	39	Five excellent rice 4	30	HS
20	Dragon japonica 1	8.2	MS	40	Loose and sticky 1	26.3	HS

## Data Availability

Data will be made available on request.

## Supplementary Materials

The following supporting information can be downloaded at: https://www.mdpi.com/article/10.3390/biology14010046/s1, Supplementary Figure S1. GIS based map of the sampling locations; Supplementary Figure S2. Liquid culture color of Ustilaginoidea virens. Note: (A), white; (B), yellow-green; (C), dark green (D), dark yellow; Supplementary Figure S3. Principal component analysis of the correlation between pathogenicity and spore production, germination rate, and liquid culture pigments; Supplementary Figure S4. Rep-PCR molecular fingerprint patterns of the genomic DNAs from some strains of Ustilaginoidea virens. Note: (A), ERIC-PCR primer amplification map; (B), BOX-PCR primer amplification map; (C), REP-PCR primer amplification map; Supplementary Table S1. GIS based map of the sampling locations; Supplementary Table S2. Evaluation criteria for resistance to Ustilaginoidea virens; Supplementary Table S3. Biological characteristics and the phenotype frequencies of biological characteristics of Ustilaginoidea virens in Heilongjiang Province.
