# Supplementary material for: Biology, Pathogenicity, and Genetic Diversity of the Rice Pathogen Ustilaginoidea virens in Heilongjiang Province, China
Source: Biology (Basel). 2025 Jan 9;14(1):46. doi: 10.3390/biology14010046 (PMC11760423; doi:10.3390/biology14010046)
Supplement: Supplementary file 1 [file biology-14-00046-s001.zip › biology-3383332-supplementary.docx]

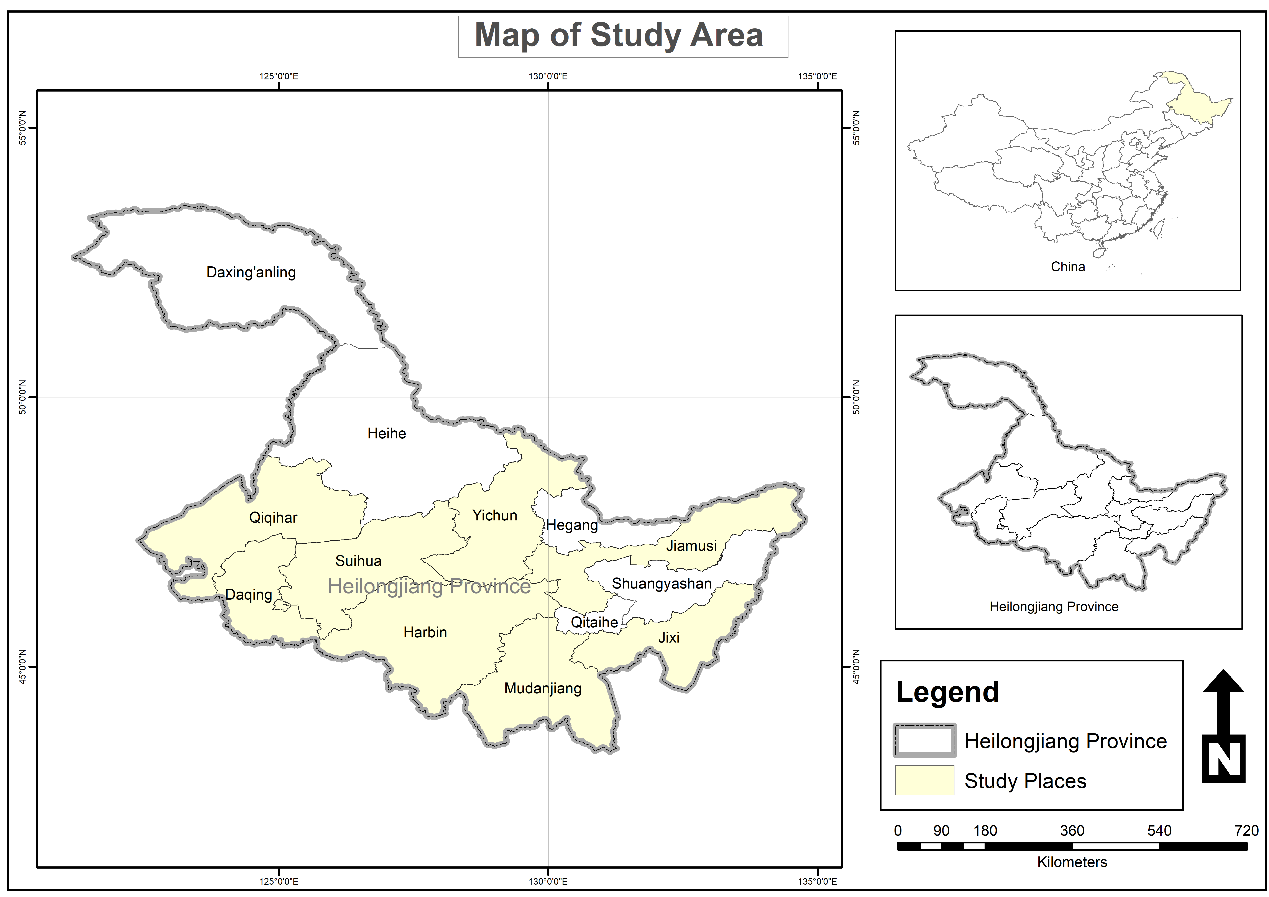


Supplementary figure 1. GIS based map of the sampling locations


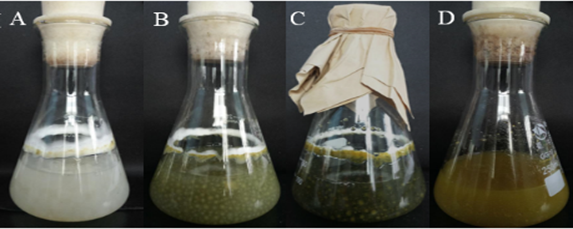


Supplementary figure 2. Liquid culture color of *Ustilaginoidea virens*

Note: (A), white; (B), yellow-green; (C), dark green (D), dark yellow


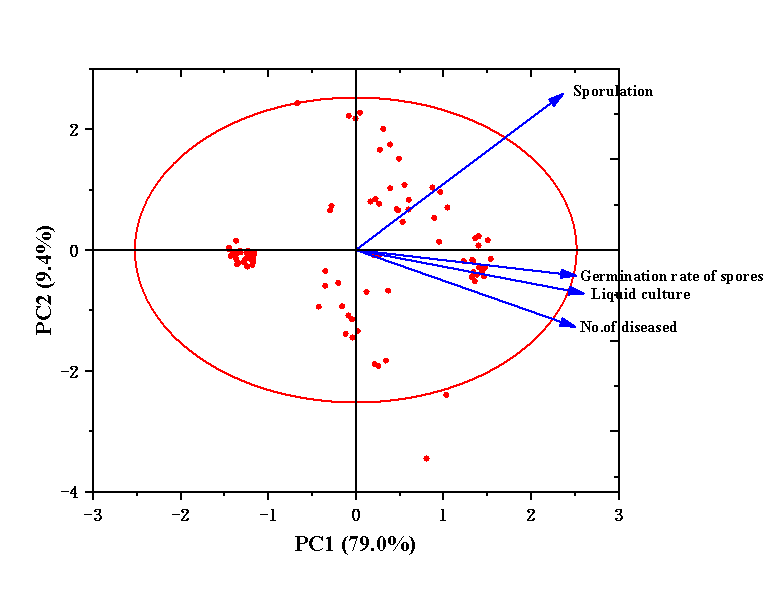


Supplementary figure 3. Principal component analysis of the correlation between pathogenicity and spore production, germination rate, and liquid culture pigments


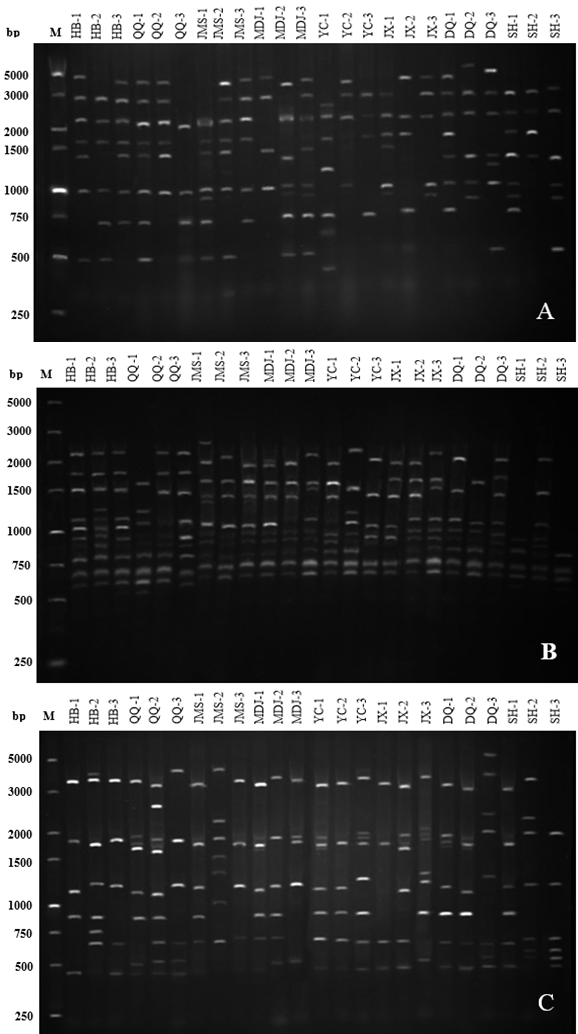


Supplementary figure 4**.** Rep-PCR molecular fingerprint patterns of the genomic DNAs from some strains of *Ustilaginoidea virens*

**Note:** **(A)**, ERIC-PCR primer amplification map; **(B)**, BOX-PCR primer amplification map; **(C)**, REP-PCR primer amplification map

Supplementary table 1. GIS based map of the sampling locations

| Strain number | Collection sites | Coordinate | Host |
| --- | --- | --- | --- |
| B-1 | Harbin | 45°45′27″N 126°38′27″E | Rice |
| B-2 | Harbin | 45°45′27″N 126°38′28″E | Rice |
| HB-3 | Harbin | 45°45′27″N 126°38′29″E | Rice |
| HB-4 | Harbin | 45°45′27″N 126°38′30″E | Rice |
| HB-5 | Harbin | 45°45′27″N 126°38′31″E | Rice |
| HB-6 | Harbin | 45°45′27″N 126°38′32″E | Rice |
| HB-7 | Harbin | 45°45′27″N 126°38′33″E | Rice |
| HB-8 | Harbin | 45°45′27″N 126°38′34″E | Rice |
| HB-9 | Harbin | 45°45′27″N 126°38′35″E | Rice |
| HB-10 | Harbin | 45°45′27″N 126°38′36″E | Rice |
| HB-11 | Harbin | 45°45′27″N 126°38′37″E | Rice |
| HB-12 | Harbin | 45°45′27″N 126°38′38″E | Rice |
| HB-13 | Harbin | 45°45′27″N 126°38′39″E | Rice |
| HB-14 | Harbin | 45°45′27″N 126°38′40″E | Rice |
| HB-15 | Harbin | 45°45′27″N 126°38′41″E | Rice |
| QQ-1 | Qiqihar | 47°21′18″N 123°55′06″E | Rice |
| QQ-2 | Qiqihar | 47°21′18″N 123°55′07″E | Rice |
| QQ-3 | Qiqihar | 47°21′18″N 123°55′08″E | Rice |
| QQ-4 | Qiqihar | 47°21′18″N 123°55′09″E | Rice |
| QQ-5 | Qiqihar | 47°21′18″N 123°55′10″E | Rice |
| QQ-6 | Qiqihar | 47°21′18″N 123°55′11″E | Rice |
| QQ-7 | Qiqihar | 47°21′18″N 123°55′12″E | Rice |
| QQ-8 | Qiqihar | 47°21′18″N 123°55′13″E | Rice |
| QQ-9 | Qiqihar | 47°21′18″N 123°55′14″E | Rice |
| QQ-10 | Qiqihar | 47°21′18″N 123°55′15″E | Rice |
| QQ-11 | Qiqihar | 47°21′18″N 123°55′16″E | Rice |
| QQ-12 | Qiqihar | 47°21′18″N 123°55′17″E | Rice |
| QQ-13 | Qiqihar | 47°21′18″N 123°55′18″E | Rice |
| JMS-1 | Jiamusi | 46°48′00″N 130°19′08″E | Rice |
| JMS-2 | Jiamusi | 46°48′00″N 130°19′09″E | Rice |
| JMS-3 | Jiamusi | 46°48′00″N 130°19′10″E | Rice |
| JMS-4 | Jiamusi | 46°48′00″N 130°19′11″E | Rice |
| JMS-5 | Jiamusi | 46°48′00″N 130°19′12″E | Rice |
| JMS-6 | Jiamusi | 46°48′00″N 130°19′13″E | Rice |
| JMS-7 | Jiamusi | 46°48′00″N 130°19′14″E | Rice |
| JMS-8 | Jiamusi | 46°48′00″N 130°19′15″E | Rice |
| JMS-9 | Jiamusi | 46°48′00″N 130°19′16″E | Rice |
| JMS-10 | Jiamusi | 46°48′00″N 130°19′17″E | Rice |
| JMS-11 | Jiamusi | 46°48′00″N 130°19′18″E | Rice |
| MDJ-1 | Mudanjiang | 44°33′05″N 129°37′58″E | Rice |
| MDJ-2 | Mudanjiang | 44°33′05″N 129°37′59″E | Rice |
| MDJ-3 | Mudanjiang | 44°33′05″N 129°37′60″E | Rice |
| MDJ-4 | Mudanjiang | 44°33′05″N 129°37′61″E | Rice |
| MDJ-5 | Mudanjiang | 44°33′05″N 129°37′62″E | Rice |
| MDJ-6 | Mudanjiang | 44°33′05″N 129°37′63″E | Rice |
| MDJ-7 | Mudanjiang | 44°33′05″N 129°37′64″E | Rice |
| MDJ-8 | Mudanjiang | 44°33′05″N 129°37′65″E | Rice |
| MDJ-9 | Mudanjiang | 44°33′05″N 129°37′66″E | Rice |
| MDJ-10 | Mudanjiang | 44°33′05″N 129°37′67″E | Rice |
| MDJ-11 | Mudanjiang | 44°33′05″N 129°37′68″E | Rice |
| MDJ-12 | Mudanjiang | 44°33′05″N 129°37′69″E | Rice |
| MDJ-13 | Mudanjiang | 44°33′05″N 129°37′70″E | Rice |
| YC-1 | Yichun | 47°43′41″N 128°50′28″E | Rice |
| YC-2 | Yichun | 47°43′41″N 128°50′29″E | Rice |
| YC-3 | Yichun | 47°43′41″N 128°50′30″E | Rice |
| YC-4 | Yichun | 47°43′41″N 128°50′31″E | Rice |
| YC-5 | Yichun | 47°43′41″N 128°50′32″E | Rice |
| YC-6 | Yichun | 47°43′41″N 128°50′33″E | Rice |
| YC-7 | Yichun | 47°43′41″N 128°50′34″E | Rice |
| JX-1 | Jixi | 45°17′42″N 130°58′08″E | Rice |
| JX-2 | Jixi | 45°17′42″N 130°58′09″E | Rice |
| JX-3 | Jixi | 45°17′42″N 130°58′10″E | Rice |
| JX-4 | Jixi | 45°17′42″N 130°58′11″E | Rice |
| JX-5 | Jixi | 45°17′42″N 130°58′12″E | Rice |
| JX-6 | Jixi | 45°17′42″N 130°58′13″E | Rice |
| JX-7 | Jixi | 45°17′42″N 130°58′14″E | Rice |
| JX-8 | Jixi | 45°17′42″N 130°58′15″E | Rice |
| JX-9 | Jixi | 45°17′42″N 130°58′16″E | Rice |
| JX-10 | Jixi | 45°17′42″N 130°58′17″E | Rice |
| JX-11 | Jixi | 45°17′42″N 130°58′18″E | Rice |
| DQ-1 | Daqing | 46°35′20″N 125°06′14″E | Rice |
| DQ-2 | Daqing | 46°35′20″N 125°06′15″E | Rice |
| DQ-3 | Daqing | 46°35′20″N 125°06′16″E | Rice |
| DQ-4 | Daqing | 46°35′20″N 125°06′17″E | Rice |
| DQ-5 | Daqing | 46°35′20″N 125°06′18″E | Rice |
| DQ-6 | Daqing | 46°35′20″N 125°06′19″E | Rice |
| DQ-7 | Daqing | 46°35′20″N 125°06′20″E | Rice |
| DQ-8 | Daqing | 46°35′20″N 125°06′21″E | Rice |
| DQ-9 | Daqing | 46°35′20″N 125°06′22″E | Rice |
| SH-1 | Suihua | 46°39′14″N 126°58′08″E | Rice |
| SH-2 | Suihua | 46°39′14″N 126°58′09″E | Rice |
| SH-3 | Suihua | 46°39′14″N 126°58′10″E | Rice |
| SH-4 | Suihua | 46°39′14″N 126°58′11″E | Rice |
| SH-5 | Suihua | 46°39′14″N 126°58′12″E | Rice |
| SH-6 | Suihua | 46°39′14″N 126°58′13″E | Rice |
| SH-7 | Suihua | 46°39′14″N 126°58′14″E | Rice |

Supplementary table 2. Evaluation criteria for resistance to *Ustilaginoidea virens*

| Susceptibility level DL | Lv. 0 | Lv. 1 | Lv. 2 | Lv. 3 | Lv. 5 | Lv. 7 |
| --- | --- | --- | --- | --- | --- | --- |
| evaluation of resistance | Immune I | Disease-resistant R | Medium anti-MR | Moderately susceptible MS | Susceptible S | High susceptibility HS |
| Ear disease rate DSR | 0% | 0.1~3% | 3.1~7% | 7.1~15% | 15.1~25% | ≥25% |
| Number of diseased kernel |  | ≤5 | ≤7 |  |  |  |

Note: The number of infected panicles was the number of the most serious panicles. When the rate of infected panicles (0.1%-3%), the number of infected panicles ≤5 was classified as grade 1; when the number of infected panicles ≥6, the grade of infected panicles was reduced to grade 3; When the head rate was (3.1-7%), the head rate was ≤7, the susceptible grade was 3,and when the number of grains was ≥8, the susceptible grade was reduced to 5

Supplementary table 3. Biological characteristics and the phenotype frequencies of biological characteristics of *Ustilaginoidea virens* in Heilongjiang Province

| **Region** | **Mycelium growth rate**  **(mm/d)** | **Sporulation**  **(10^4^/mL)** | **Germination rate**  **of spores**  **(%)** | **No.of diseased grain/panicle** | **Growth rate** | | | **Sporulation** | | | **Spore germination ability** | | | **Liquid culture pigment** | | | | **Pathogenicity** | | |
| --- | --- | --- | --- | --- | --- | --- | --- | --- | --- | --- | --- | --- | --- | --- | --- | --- | --- | --- | --- | --- |
| Region |  |  |  |  | Fast | Medium | Slow | Much | Medium | Little | Strong | Medium | Weak | White | Yellow green | Atrovirens | Yellow | Strong | Medium | Weak |
| Harbin | 2.46 | 210.13 | 58.07 | 13.27 | 40% | 33% | 27% | 60% | 20% | 20% | 33% | 40% | 27% | 27% | 27% | 27% | 20% | 27% | 47% | 27% |
| Qiqihar | 2.42 | 138.15 | 53.08 | 12.84 | 31% | 38% | 31% | 38% | 31% | 31% | 31% | 38% | 31% | 31% | 38% | 23% | 8% | 23% | 46% | 31% |
| Jiamusi | 2.01 | 163.09 | 62.82 | 12.19 | 27% | 9% | 64% | 36% | 36% | 27% | 45% | 27% | 27% | 27% | 18% | 27% | 27% | 27% | 45% | 27% |
| Mudanjiang | 2.28 | 132.92 | 54.38 | 13.22 | 23% | 38% | 38% | 23% | 46% | 31% | 23% | 46% | 31% | 31% | 46% | 8% | 15% | 23% | 46% | 31% |
| Yichun | 2.45 | 131.57 | 54.43 | 12.01 | 29% | 43% | 29% | 29% | 29% | 43% | 29% | 29% | 43% | 43% | 43% | 14% | 0% | 29% | 29% | 43% |
| Jixi | 2.45 | 124.00 | 62.00 | 16.35 | 27% | 36% | 36% | 18% | 55% | 27% | 36% | 36% | 27% | 27% | 18% | 18% | 36% | 36% | 36% | 27% |
| Daqing | 3.18 | 167.33 | 55.44 | 19.02 | 67% | 22% | 11% | 56% | 11% | 33% | 44% | 22% | 33% | 33% | 0% | 22% | 44% | 44% | 22% | 33% |
| Suihua | 2.56 | 137.71 | 53.43 | 9.76 | 57% | 0% | 43% | 43% | 14% | 43% | 29% | 29% | 43% | 43% | 29% | 14% | 14% | 14% | 43% | 43% |
